# Development of Lead-Free Radiation Shielding Material Utilizing Barium Sulfate and Magnesium Oxide as Fillers in Addition Cure Liquid Silicone Rubber

**DOI:** 10.3390/polym15224382

**Published:** 2023-11-10

**Authors:** Everton G. Souza, Kaiser Kruger, Chiara D. Nascimento, Cesar Aguzzoli, Gabriela Hoff, Ana Cristina B. K. Moraes, Rafael G. Lund, Patrícia S. Nascente, Carlos E. Cuevas-Suárez, Evandro Piva, Neftali L. V. Carreno

**Affiliations:** 1 Graduate Program in Electronic and Computer Engineering, Catholic University of Pelotas, Pelotas 96015-560, Brazil; kaiser_kruger@yahoo.com.br (K.K.); chiara.nascimento@ucpel.edu.br (C.D.N.); 2 Graduate Program in Materials Science and Engineering, University of Caxias do Sul, Caxias 95070-560, Brazil; caguzzol@ucs.br; 3 Medical Physics and Radioprotection Service, Clinical Hospital of Porto Alegre, Porto Alegre 90035-903, Brazil; ghoff.gesic@gmail.com; 4 School of Dentistry, Federal University of Pelotas, Pelotas 96010-560, Brazil; ana.moraes@ucpel.edu.br (A.C.B.K.M.); rglund@ufpel.edu.br (R.G.L.); piva@ufpel.edu.br (E.P.); 5 Graduate Program in Materials Science and Engineering, Technology Development Center, Federal University of Pelotas, Pelotas 96010-610, Brazil; neftali@ufpel.edu.br; 6 Biology Institute, Federal University of Pelotas, Pelotas 96160-000, Brazil; patricia.nascente@ufpel.edu.br; 7 Department of Dentistry Surgery, Autonomous University of Hidalgo, Pachuca de Soto 42080, México; cecuevas@uaeh.edu.mx

**Keywords:** ionizing radiation, lead-free elastomeric radiological protection, barium sulfate filler, magnesium oxide filler, addition cure liquid silicone rubber, Geant4 Monte Carlo toolkit, transmitted radiation

## Abstract

The radiological protection has the purpose of safeguarding the physical well-being of the user, preventing exposure to detrimental levels of ionizing radiation. This study introduces a novel, cost-effective category of lead-free elastomeric material designed for radiation shielding. The filler compounds utilized are notably lighter than conventional lead-based materials, enhancing user ergonomics during application. They comprise of a blend of barium sulfate combined or not with magnesium oxide with addition-cure liquid silicone rubber. To ensure the effectiveness of the radiation shielding, X-ray transmission measurements were performed for the different thicknesses of the materials and the results compared with Monte Carlo simulations. Additionally, the physical properties of the new materials, such as density, homogeneity, tensile strength, viscosity, and wettability, were also evaluated. The findings indicate that both materials fulfill the requirement for application in radiation protection garments.

## 1. Introduction

In countries with a developed clinical sector, up to a further 50% of the radiation exposure is attributed to medical sources [1]. Thus, for dose limitation purposes, the International Commission on Radiological Protection (ICRP) has created effective dose limits aiming to reduce the risks of stochastic effects [2]. In addition to these limits, taking into account a conservative application of radiological protection, one can consider that there is no safe dose of radiation exposure since hereditary effects are not considered in these studies [3,4].

To minimize exposure and control the dose in radiodiagnostic exams, it is necessary for both patients and workers to use personal radiation protective equipment (PRPE) in healthcare facilities during X-ray procedures. This includes wearing lead aprons, gloves, masks, and gonadal and thyroid shields, along with protective eyewear [5].

Historically, PRPE has been manufactured using lead powder-loaded polymers and low-melting-point lead alloys [6,7]. The heavy weight [8,9,10], low flexibility [11], fragility [12], and toxicity [13] of the shielding materials are described as the main technological challenges for the development of safe and top-quality garments. Progress has been achieved with the incorporation of metal powders into polymer sheets, increasing the compound resistance against cracks and other forms of deterioration [14,15]. The weight of the shielding garments and the lead content has been minimized with the replacement of a fraction of lead with new elements with a moderate atomic number such as tin, antimony, and barium [16,17].

Scuderi et al. [18] suggested radiological protective clothing based on tin, arsenic, and cadmium, while other authors [19,20,21,22,23] recommended the usage of composite materials with tin, tantalum, and tungsten as alternatives to lead. Although the authors have reported advantages such as low weight and reasonable shielding, flexibility problems compromised the quality of the compounds due to the addition of tin. Moreover, tin and cadmium play no known natural biological role in living organisms [24], and long-term exposure to arsenic results in chronic poisoning (arsenicosis) [25].

In previous investigations [17,26], barium was employed in combination with other heavy metal powders, including bismuth and tungsten, along with the renewable biopolymer amorphous cellulose; these were used as additives within a high-viscosity silicone rubber (SR) matrix. Some of these studies [27] involved the incorporation of substantial loading levels, reaching a weight ratio of 60%, into the polymer matrix. While the authors asserted that there were no substantial modifications in the physical properties of the composite material, it is noteworthy that no comprehensive characterization measurements were presented, despite the potential for such a significant quantity of additives to affect these properties.

The main objective of this investigation is to introduce a novel, cost-effective, and easily processable lead-free elastomeric radiation shielding material intended for use in radiation shielding garments. This material incorporates barium sulfate (BaSO_4_), chosen over barium due to its superior density; lower cost; and, consequently, enhanced shielding capabilities [28]. BaSO_4_ is well-tolerated by the human body and has been widely used in medical applications as a radio-contrast agent for X-ray imaging due to its radiopaque properties [29]. Additionally, it has been employed in radiation shielding applications for materials such as concrete when combined with fly ash geopolymers (FAGP) [30,31,32,33], as well as in skin protection creams [34].

However, BaSO_4_ tends to agglomerate when mixed with liquid silicone rubber, resin, and rubber. This process leads to the uneven distribution of the product, resulting in voids that can compromise the shielding quality [35,36]. To address these issues, the use of nanoscale barium particles [17] or dispersant agents [37] can aid in achieving a more uniform distribution of the materials. This, in turn, reduces particle aggregation and minimizes the occurrence of voids.

Our approach investigates the incorporation of BaSO_4_ and BaSO_4_ with magnesium oxide (MgO), a dispersant agent for BaSO_4_, into an addition-cure liquid silicone rubber (ALSR) as the polymeric matrix, deviating from conventional condensation cure systems such as silicone rubber (SR) or liquid silicone rubber (LSR) [22]. For our analysis, samples containing ALSR with 10% BaSO_4_ and ALSR with 10% BaSO_4_ with an additional 10% MgO have undergone measurements of wettability, density, viscosity, morphology, and radiation shielding effectiveness.

To establish a better correlation between the experimental findings and theoretical models, a simplified geometry consisting of a single source, one ionization chamber, and one scatterer material were performed with Geant4 Monte Carlo toolkit [38,39].

The work was structured as follows: Section 2 provides a description of the experimental procedures for manufacturing the ALSR samples with BaSO_4_ (ALSR-Ba) and with BaSO_4_ and MgO (ALSR-Ba-Mg), the technical specifications of the X-ray equipment, the details of the experimental setup used for irradiation tests, and the characterization measurements of the samples. In Section 3, we present the results of the mechanical, morphological, and surface measurements of the analyzed samples. This section also introduces a virtual environment utilizing the Geant4 Monte Carlo toolkit to compare the shielding effectiveness of the materials with the experimental results. Finally, in Section 4, we present the paper’s conclusions.

## 2. Materials and Methods

### 2.1. Manufacture of the Samples

In the sample manufacturing process, the initial compound, referred to as ALSR-Ba, was formulated with a mass ratio of 90.00% ALSR (poly(dimethylsiloxane)-PDMS) having a shore hardness of 14 (Siquiplás, model 6014, Santana, SP, Brazil), combined with 10.00% BaSO_4_ (barium sulfate) in powder form, obtained from NEON Comercial Reagentes Químicos Ltda, Suzano, SP, Brazil, with a purity of 98.46%.

The ALSR and the powdered additive were manually mixed in a beaker for a duration of 5 min. The purpose of this mixing process was to enhance the dispersion of the additives and achieve a more homogeneous mixture. During the final minute of mixing, 5% of the total mass of organotin catalyst (dibutyltin dilaurate), with a purity of 95%, was added to expedite the curing process. The resulting mixture was immediately poured into custom-made molds, created using 3D printing technology, with dimensions of 10 cm × 10 cm and varying thicknesses of 1 mm, 2 mm, 3 mm, 4 mm, 8 mm, and 12 mm, aiming the irradiation tests. The manufactured compound was then left rest for 24 h until it was cured and reached a solid form.

The ALSR-Ba-Mg compound was synthesized using the same manufacturing recipe, with the addition of MgO during the initial stage. The resulting percent composition of the compound was 80.00% ALSR, 10.00% BaSO_4_, and 10.00% MgO (LabSynth Produtos para Laboratórios Ltda, Diadema, SP, Brazil, purity 98.0%).

### 2.2. Sample Characterizations

#### 2.2.1. Tensile Tests

For the mechanical characterization, uniaxial tensile tests were conducted using a Tensile Testing Machine (Emic, model DL-500, São José dos Pinhais, PR, Brazil) with a constant speed of 12.5 mm/min and a load cell capacity of 1000 N. Prior to the tests, the samples underwent a 2-h conditioning period at a temperature of 25 °C and a relative humidity of 50%, in accordance with ASTM D412 standard [40].

**Figure 1:**
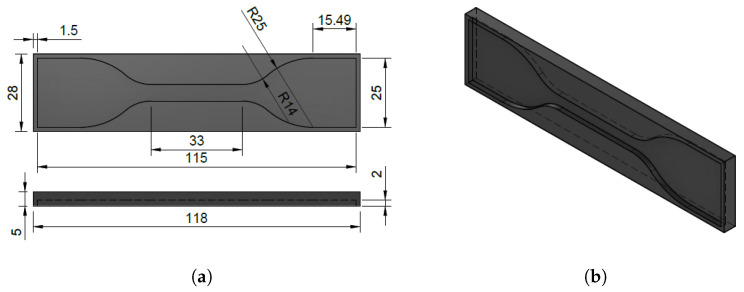
(**a**) Top view and (**b**) 3D view of 3D printing custom-made molds manufactured according to ASTM D412 [40]. All the dimensions as expressed in mm. The internal volume of the mold amounts to 3.627 cm^3^.

Following the guidelines outlined in Section 2.1, the ALSR mixture combined with additive powders was poured in liquid state into custom-made molds with dumbbell shapes, created using 3D printing technology. The dimensions and formats of the molds comply with the ASTM D412 standard and can be observed in Figure 1, frames (a) and (b).

In this experiment, the samples were prepared in triplicate for each of the three compound types: ALSR-Ba, ALSR-Ba-Mg, and pure silicone, with the objective of evaluating the fluctuations in tensile strength.

#### 2.2.2. Wettability

In order to assess the wettability characteristics of the samples, the sessile drop technique, with a drop volume of 10 μL, was employed using a optical tensiometer (theta life, model TL100, manufactured by Biolin Scientific, Gothenburg, Sweden). The technique involved generating three micrometer-scale droplets of distilled water for each sample and subsequently measuring each droplet at five distinct locations of each specimen. The average contact angle was determined using the image analysis program, OneAttension.

#### 2.2.3. Density

The densities were measured using a precision immersion technique. Triplicate samples of pure ALSR, ALSR-Ba, and ALSR-Ba-Mg compounds were weighed on an analytical balance (Quimis, BG 400, Diadema, SP, Brazil) and submerged in distilled water held in a 25 mL beaker (Pyrex, 25 mL, 0.05 mL precision). The temperature and pressure of the water were monitored and maintained at 25 °C, with a relative humidity of 66% and atmospheric pressure, respectively. The Archimedes’ principle was applied to calculate the density of each compound. The obtained density values were analyzed for accuracy and consistency, ensuring the reliability of the measurements.

#### 2.2.4. Viscosity

The viscosity of ALSR, ALSR-Ba, and ALSR-Ba-Mg was determined using a rotary rheometer (RS-CPS+, Brookfield Engineering Laboratories, Middleborough, MA, USA) to perform rheological measurements. A volume of 0.5 mL of each material (ALSR, ALSR-Ba, and ALSR-Ba-Mg) without the catalyst was placed on the bottom plate of the rheometer. The upper plate, with a diameter of 25 mm, was positioned at a distance of 0.05 mm from the bottom plate. Viscosity (Pa.s) was measured for 90 s with 30 data points, using a constant shear rate of 100 s^−1^ and an average temperature of 20.5 °C. The rheological parameters were determined and calculated using the Rheo 3000 software version 1.2.

#### 2.2.5. Morphology

Scanning electron microscopy (SEM) was employed to evaluate the morphology of 1.0 × 1.0 × 0.2 cm-sized samples using the TESCAN VEGA3 model, operating at 20 kV with a magnification of 500×. The uniformity of the additive powders within the silicone matrix was appraised through energy dispersive spectroscopy (EDS), which was integrated with SEM using the Bruker Nano XFlash Detector 6-10 for chemical mapping.

#### 2.2.6. Irradiation Tests

In order to evaluate the radiation absorption properties, primary beams with an exposure time of 20 ms; a current of 200 mA; and peak tensions of 50, 60, and 80 kVp were generated using a digital X-ray equipment (Siemens, model Axion Iconos, number 0483404). Before each shot, for ensuring reproducibility, the incident beam was collimated exactly with the same size of the detector area, which was completely covered by the absorbing materials, following a similar procedure as suggested by Scaff [41].

The primary X-ray beam was initially directed towards the X-ray QA meter (RTI, model Black Piranha), located at a source-to-surface distance (SSD) of 115.0 cm, in order to measure the intensity of the direct beam without any shielding. To prevent scattered radiation at the collimator exit, a 1 mm lead mask with a window area matching that of the irradiated material was employed.

**Figure 2:**
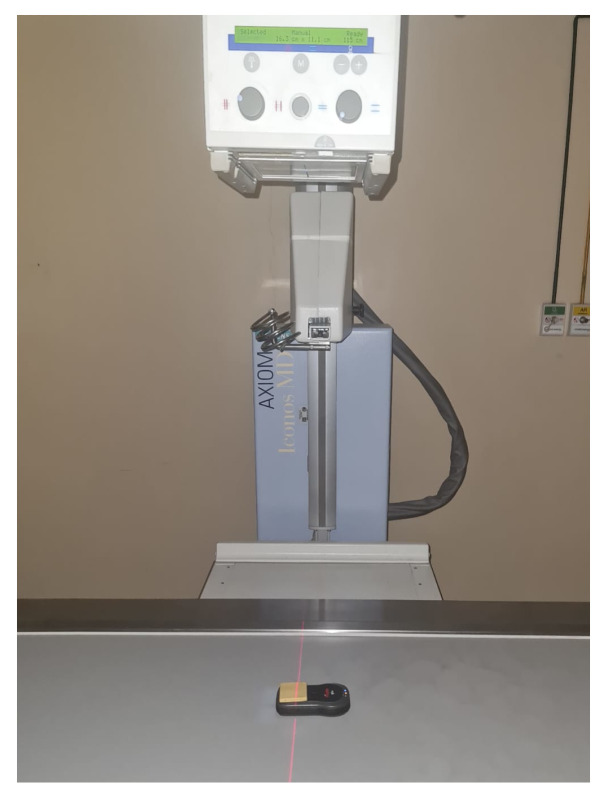
X-ray exposure depicting the evaluation of shielding effectiveness using a 4 mm sample placed on the X-ray QA meter.

Following that, the samples were placed onto the X-ray QA meter, with one sample used for each X-ray exposure. This process was repeated six times, with varying thicknesses, for each kVp value. The objective was to evaluate the shielding effectiveness in relation to the thickness of the samples. Figure 2 illustrates one of these exposures, specifically utilizing a 4 mm sample. Both the energy range and thickness values were imposed by the physical characteristics of the materials, which were chosen according to previous knowledge in preliminary tests.

## 3. Results and Discussion

### 3.1. Material Characterization

**Figure 3:**
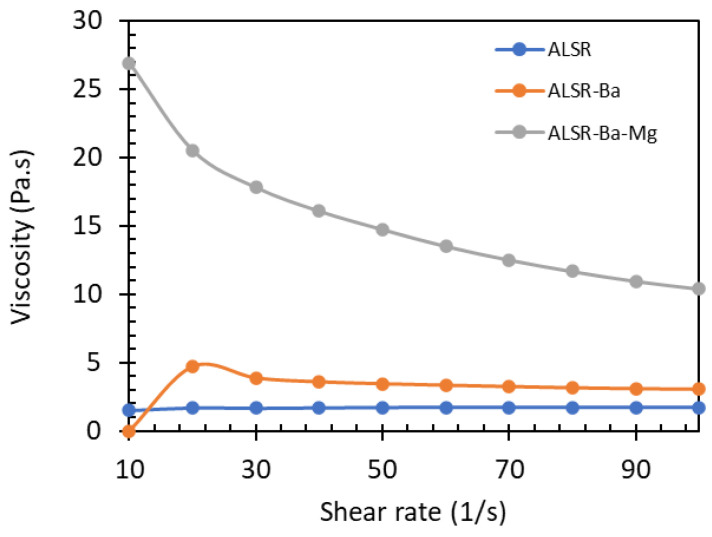
Viscosity values for different shear rate for the ALSR 6014.

The ability of an addition-cure liquid silicone to maintain controlled and adaptable viscosity across a broad range of shear rates is paramount for its versatility in molding molds of varying thicknesses. This plays a pivotal role in the efficiency of the manufacturing process and ensuring the production of high-quality shielding products, as depicted in Figure 3.

The presented results indicate non-Newtonian behavior, characterized by the viscosity of the liquid silicone varying with the shear rate. While there is a decrease in viscosity as the shear rate increases—which is more prominent in ALSR-Ba-Mg and ALSR-Ba, the materials with higher viscosity—the magnitude of this variation remains relatively constant across the tested range of shear rates, especially for ALSR. This suggests a controlled and relatively consistent viscosity. This implies that the liquid silicone can evenly fill molds with varying thicknesses and designs, playing a crucial role in ensuring consistent shielding for different types of molds.

When ALSR is mixed with powder additives as BaSO_4_ and MgO, both powders can act as reinforcing fillers in the silicone rubber mixture, changing its the fundamental characteristics. Table 1 shows the results of the density and contact angle for each of the three compound studies in this work: ALSR, ALSR-Ba, and ALSR-Ba-Mg.

Enhancing the resultant compound’s density, as well as the shielding efficiency, can be achieved by incorporating a precisely controlled proportion of additive powder, 10% of BaSO_4_ with 90% of ALSR for ALSR-Ba and 10% of BaSO_4_, and 10% of MgO with 80% of ALSR for ALSR-Ba-Mg. The obtained density values, 1.80 (0.09) and 1.83 (0.03), for which the differences were not statistically significant, consistently align with the respective fractions of each element. In the case of pure powders, the densities recorded were 3.50 (0.05) g/cm${}^{3}$ for BaSO_4_ and 1.90 (0.10) g/cm${}^{3}$ for MgO.

In terms of sample wettability, ALSR without the addition of powder filler exhibited an 106.31° contact angle, indicating a moderately hydrophobic surface. This suggests that pure liquid silicone has some affinity for water but is not highly repellent. This contact angle value is in agreement with that reported by [42] for ALSR based on fluorine-containing polysiloxane low-melting glass with tridecafluorooctyltriethoxysilane (FAS).

Differences among the contact angle were not statistically significant (p=0.159). The addition of 10% BaSO_4_ to the liquid silicone slightly augmented the contact angle to 110.32°, whereas the incorporation of 10% MgO with 10% BaSO_4_ into the liquid silicone further elevated the contact angle to approximately 115.17°. This outcome is primarily attributed to the low solubility of MgO and BaSO_4_ in water. It also correlates with the amount of filler added to the ALSR; as in the ALSR-Ba-Mg sample, 20% of the total mass consists of a sparingly water-soluble filler, whereas in the ALSR-Ba sample, it contains only 10% of BaSO_4_. This aspect is quite advantageous as the increase in surface hydrophobicity retards the deposition of contaminants.

**Figure 4:**
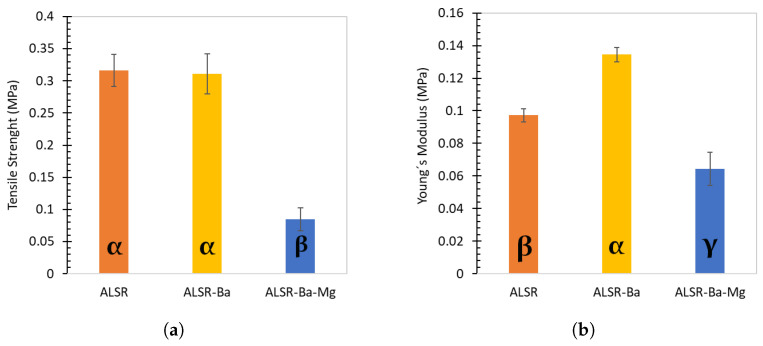
Mean and standard deviation of the tensile strength (**a**) and Young’s modulus (**b**) for the three formulations studied: pure silicone, ALSR-Ba, and ALSR-Ba-Mg. The error bars displayed correspond to the standard deviation associated with measurements obtained from three identical test specimens. The means indicated by the same greek letter do not differ significantly from each other.

Regarding the mechanical strength of the samples, the additions of barite and magnesium oxide have a statistically significant influence on the mechanical properties of the silicone rubbers (p<0.001), as illustrated in Figure 4, panels (a) and (b).

As illustrated in Figure 4a, pure silicone exhibits the highest tensile strength (0.3164 MPa), while the formulation with BaSO_4_ slightly diminishes this value (0.3107 MPa); however, the differences were not statistically significant (p=0.9654). The formulation containing BaSO_4_ + MgO significantly reduces tensile strength (0.0848 MPa), indicating a negative impact of magnesium oxide addition on strength (*p* < 0.001).

Regarding Young’s modulus, as depicted in Figure 4b, it serves as a measure quantifying material stiffness. In this context, the formulation with BaSO_4_ possesses the highest Young’s modulus (0.1345 MPa), indicating greater stiffness than pure silicone (0.0973 MPa). Conversely, the formulation with barite mixed with magnesium oxide exhibits the lowest Young’s modulus (0.0642 MPa), signifying reduced stiffness.

Therefore, the BaSO_4_ formulation demonstrates an advantageous combination of stiffness (high Young’s modulus) and tensile strength similar to pure silicone. This crucial property is advantageous in radiological applications, as we will discuss further, where BaSO_4_ outperforms pure silicone significantly in X-ray shielding. Conversely, MgO reduces silicone stiffness while rendering it more brittle.

The analysis of ALSR’s mechanical properties assumes a fundamental role in comprehending its handling, durability, elasticity, and rigidity. However, within the context of radiation shielding, density emerges as a critical parameter as denser materials typically yield enhanced radiation attenuation capabilities. Nevertheless, for dense materials to effectively attenuate radiation, homogeneity must be ensured throughout their entire thickness. Non-homogeneity may result in the transmission of unattenuated radiation through less dense areas, thereby compromising the shielding effectiveness and increasing the risk of exposure to hazardous radiation.

**Figure 5:**
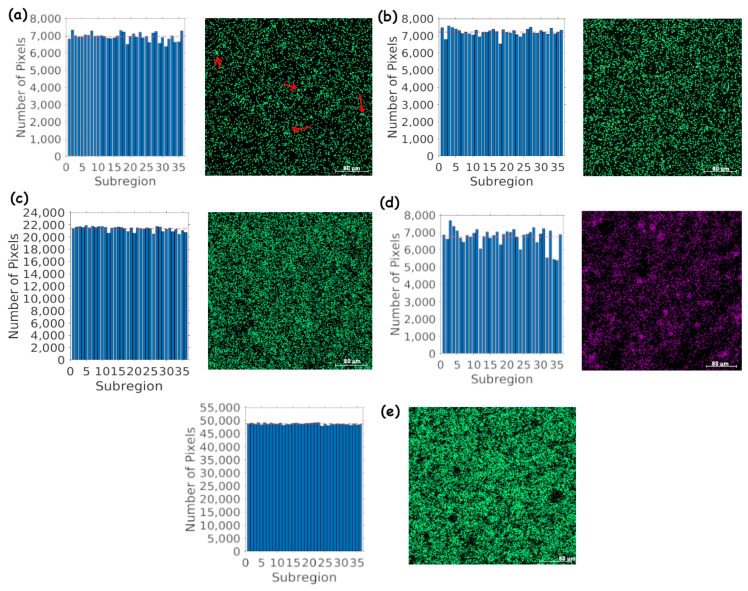
Homogeneity analysis with EDS in ALSR with varying proportions of BaSO_4_ and MgO, using energy-dispersive spectroscopy (EDS). ALSR with: (**a**) 10% BaSO_4_, (**b**) ALSR with 10% BaSO_4_ (combined with 10% MgO), (**c**) 30% BaSO_4_, (**d**) 10% MgO (combined with 10% BaSO_4_), and (**e**) 50% BaSO_4_. The scale bar in the lower right corner of the SEM image represents a length of 80 μm, and the image was magnified 500 times. The red arrows indicate clusters of barium.

Energy dispersive spectroscopy (EDS) is an effective analytical technique for investigating the elemental composition of materials, as well as their distribution within a sample. In this context, materials containing ALSR and varying proportions of BaSO_4_ and MgO were examined, and the results were compared through a composition map histogram. The specimens with 10% BaSO_4_ (ALSR-Ba) and 10% BaSO_4_ with 10% MgO (ALSR-Ba-Mg) are the ones that have been subject to examination in the preceding measurements. Figure 5 illustrates the results.

In Figure 5a, representing ALSR with 10% BaSO_4_, the histogram and composition map demonstrate a homogeneous distribution of BaSO_4_ within the silicone matrix. However, it is important to note that the more pronounced oscillation in the histogram is due to the lower percentage of BaSO_4_ used, which can result in a more visible variation in the quantity of barium atoms concerning the midline. This variation, although apparent in the graph, still indicates a relatively uniform distribution of BaSO_4_, with the formation of small clusters of barium (as indicated by the red arrows) in a limited proportion. These clusters can arise due to the inherent challenge of achieving an entirely uniform dispersion of barite within the silicone matrix, especially when a relatively low percentage of BaSO_4_ is utilized.

On the other hand, in Figure 5b, illustrating ALSR with 10% BaSO_4_ and 10% MgO, homogeneity is more pronounced, even though large clusters of MgO are present, as shown in Figure 5d. The effectiveness of MgO as a dispersing agent is evident here. Although MgO also forms clusters, BaSO_4_ is more uniformly dispersed within the silicone matrix. This occurs because MgO acts as an agent that assists in a more even dispersion of BaSO_4_, reducing the formation of BaSO_4_ clusters. Figure 5b confirms this effectiveness, emphasizing the uniformity in the distribution of barium within the silicone matrix.

In contrast, in Figure 5c,e, representing ALSR with 30% and 50% barite, respectively, not only is the quantity of barium atoms higher but also the formation of clusters is virtually nonexistent, indicating greater ease in dispersing barite uniformly as the concentration increases. The result also indicates that MgO is more crucial in scenarios involving reduced amounts of BaSO_4_.

### 3.2. Montecarlo Simulations and Effecttive Transmission Analysis

**Figure 6:**
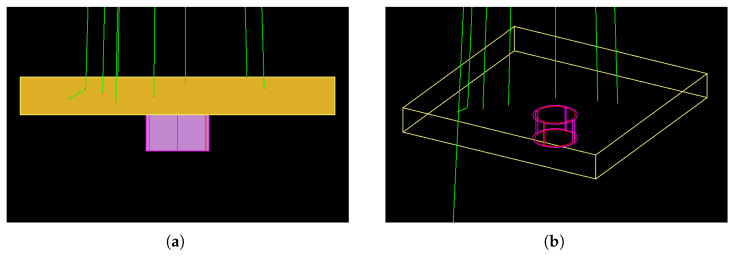
The simulated geometry was visualized using HepRep visualizer, generating an illustration comprising two perspectives: (**a**) a lateral view and (**b**) a 30° angled view. Within this visualization, distinct elements were distinguished: the scatterer (ALSR-Ba or ALSR-Ba-Mg), depicted as a yellow rectangle; the sensitive volume, depicted as a red cylinder; the external cover of the ionization chamber, depicted as a pink cylinder; photons, represented by green lines; and secondary electrons, denoted as red dots.

To establish a modeling framework for comparison with the experimental shielding results, simulations were conducted utilizing the Geant4 Monte Carlo toolkit (version 10.5.p01) [43,44,45]. The simulations were designed with a simplified geometry, incorporating a solitary source, one ionization chamber, and one scatterer material (see Figure 6).

**Figure 7:**
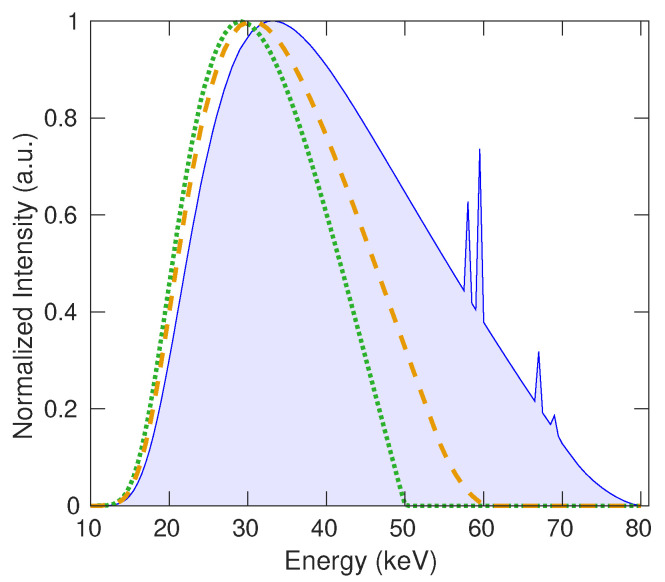
Simulated spectra emitted by the source with no filter. The dotted green line, the dashed yellow line, and the solid blue line represent the polychromatic spectra at 50, 60, and 80 kVp, respectively.

The point source that emits, homogeneously, a polychromatic spectra using the same voltage values planned for the upcoming experiment. Specifically, the voltage values utilized are 50, 60, and 80 kVp, as illustrated in Figure 7.

The X-ray beam follows a conical geometry, encompassing a maximum solid angle of 1.7°, originating from the point source. In accordance with the manufacturer’s specifications for the X-ray tube, the generation of spectra employed a 10% ripple (based on wave rectification system) and a tilt angle of 12°. These polychromatic spectra were obtained from a catalog [46] and underwent a total filtration, combining inherent and additional filtration, consisting of 2 mm of aluminum. This filtration thickness was determined deterministically using thin-layer theory.

All measurements related to the total energy deposition in the sensitive volume and the photon flux at the entrance surface of the sensitive volume were simulated using Geant4 primitive scorers (G4ScoringManager class). In this particular geometry, an ionization chamber with a volume of 6 cc was positioned at a distance of 100 cm from the radiation source. The total energy deposited in the ionization chamber was recorded by a cylindrical sensitive volume with dimensions of 0.9 cm in radius and 1.15 cm in height, positioned freely in the air. The photon flux, with energy bins of 1.0 keV, was measured by a circular surface with a radius of 0.9 cm. Additionally, a homogeneous mixture layer of each scatterer (ALSR-Ba and ALSR-Ba-Mg) was placed on top of the ionization chamber.

The Livermore low-energy model was utilized to transport both primary and secondary photons, as well as secondary electrons, with a default cut energy of 250 eV. A total of 10${}^{7}$ histories were simulated to achieve a maximum statistical fluctuation of 1.5% on the total energy deposition, with the reference value being the measurement collected without any scatterer present.

Table 2 displays the simulated composition for ALSR-Ba and ALSR-Ba-Mg used in the simulations. By disregarding more complex structural details, the basic structure of a silicone polymer can consist of repeating units with silica functional groups (Si-O) connected by covalent bonds, forming a stable three-dimensional network. The polymer in question is a polidimethylsiloxane (PDMS), also known as dimethyl silicone, which is a polymeric chain in which silica functional groups (Si-O) and methyl groups (CH_3_) alternate. Consequently, when simplifying the description of liquid silicone rubber, it is possible to consider it as a molecule composed of a basic unit represented by [Si(CH_3_)_2_-O]_n_.

For each polychromatic spectrum, simulations were conducted with different material thicknesses: 1, 2, 3, 4, 5, 8, and 12 mm, to assess the attenuation properties of the materials.

### 3.3. Radiation Shielding Effectiveness

**Figure 8:**
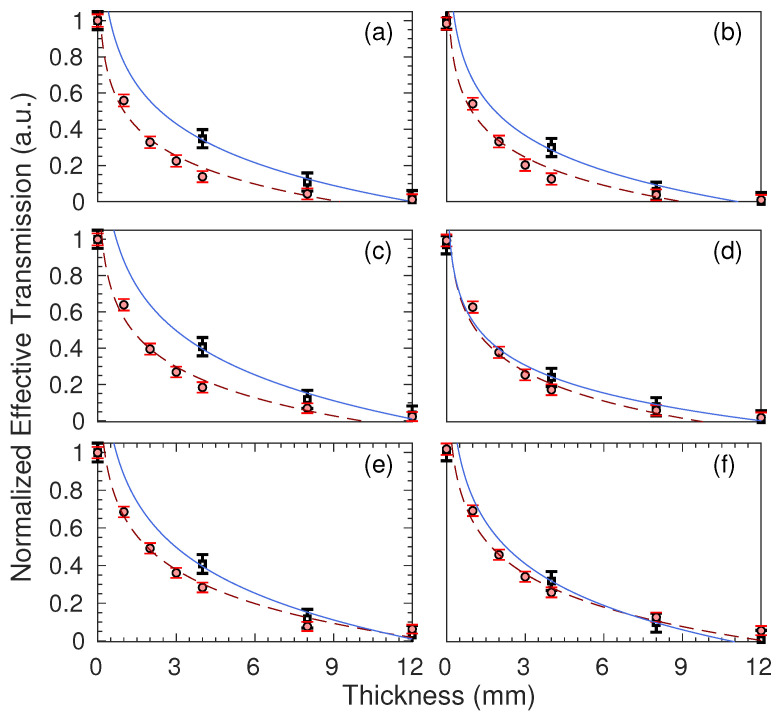
The behavior of the normalized effective transmission against the sample thicknesses. The frames (**a**,**c**,**e**) represent the ALSR-Ba compound for peak tensions values of 50, 60, and 80 kVp, respectively. The frames (**b**,**d**,**f**), in that order, represent the compound ALSR-Ba-Mg for the same peak tension values. The solid blue line represents a bivariate fit of the experimental data, while the dashed red line represents a bivariate fit of the simulated data.

The evaluation of radiation shielding effectiveness involved the analysis of normalized effective transmission for each outgoing spectrum emitted from the shielding materials. The normalized effective transmission, represented as *T*, was determined by calculating the ratio between air kerma with shielding ($K_{i}$) and air kerma without shielding ($K_{0}$), as per the relationship, $T=K_{i}/K_{0}$ [38,47]. In this investigation, $K_{i}$ is considered equivalent with the absorbed energy measured by the ionization chamber. To simplify the analysis, all of the transmission values were normalized to their respective maximum values, and the outcomes are presented in Figure 8.

Each black hollow square represents the average of five X-ray exposures (experimental data), while solid red dots depict the outcomes of Monte Carlo simulations. Error bars denote the standard deviation. Upon visual examination, in both compositions, simulations approximate the experimental data more closely as the X-ray kVp increases. The fluctuations are primarily ascribed to the utilization of a simplified composition for the liquid silicone rubber. Another potential source of error in the experimental data may be the unwanted heating of the X-ray tube due to excessive exposure on the same day. The precise disparities between the experimental measurements and the Monte Carlo-simulated data are quantified using the mean squared error (MSE), as summarized in columns 2 and 3 of Table 3.

Columns 4 and 5 present experimental radiation shielding data for a thickness of 12 mm, which corresponds to the maximum thickness examined. Under the most energetic conditions, specifically, with a primary beam featuring a 20 ms exposure time, a current of 200 mA at 80 kVp resulted in an attenuation of 95.36% for ALSR-Ba. This level of attenuation aligns with radiation shielding standards [2,48]. Notably, less energetic scenarios exhibit even greater efficiency for the same thickness. In order to provide a comparative scenario, columns 6 and 7 offer the lead equivalent for thicknesses of 0.42 mm and 0.50 mm, which are in accordance with [38].

The addition of MgO to the liquid silicone composition with BaSO_4_ loading resulted in more effective radiation shielding compared to the composition containing only BaSO_4_, with improvements ranging from 1.0% to 3.0% depending on the kVp value used. This suggests that MgO plays a crucial role in enhancing the shielding effectiveness of the composite. We believe that this enhancement is primarily attributed to MgO’s ability to influence the microstructure of the composite material, leading to a more homogeneous distribution of BaSO_4_ particles within the silicone matrix, considering that the density of both compounds is nearly identical.

However, it is important to note that MgO significantly reduces the tensile strength of ALSR. Therefore, its application should be limited to small quantities, less than 10% of the total mass, in situations where the material will not be subjected to tension.

## 4. Conclusions

In this study, we introduced two innovative lead-free radiation shielding materials consisting of an addition-cure liquid silicone rubber (ALSR) matrix filled with either barium sulfate (BaSO_4_) or a combination of BaSO_4_ and magnesium oxide (MgO).

The materials underwent comprehensive characterization, including an assessment of their physical properties, such as density and viscosity, as well as mechanical properties, encompassing tensile strength and Young’s modulus. Additionally, surface properties, specifically wettability, were examined, and their morphology was analyzed for homogeneity using EDS.

To validate their radiation shielding performance against experimental data, we conducted simulations employing the Geant4 Monte Carlo toolkit, which exhibited strong agreement with the experimental results.

In summary, both materials exhibited a shielding effectiveness exceeding 95% with a 12 mm thickness at peak tensions of 80 kVp and 4 mAs, ranging from 0.42 to 0.5 mm of Pb, which is the reference thickness used in personal radiation protective equipments.

## Figures and Tables

**Table 1:** Density, viscosity, and contact angles for samples of ALSR, ALSR-Ba, and ALSR-Ba-Mg. The quantity in parenthesis represents the uncertainty. For each column, different superscript letters indicate the presence of statistically significant differences (*p* < 0.05).

Material	Density (g/cm${}^{3}$)	Contact Angle (°)
ALSR	$1.60\;\left(0.10\right){\;}^{\beta }$	$106.31\;\left(5.11\right){\;}^{\alpha }$
ALSR-Ba	$1.80\;\left(0.09\right){\;}^{\alpha }$	$110.32\;\left(1.80\right){\;}^{\alpha }$
ALSR-Ba-Mg	$1.83\;\left(0.03\right){\;}^{\alpha }$	$105.17\;\left(6.37\right){\;}^{\alpha }$

**Table 2:** Chemical composition of ALSR-Ba and ALSR-Ba-Mg compounds adopted for the simulations.

Components	Material	
	ALSR-Ba (% wt)	ALSR-Ba-Mg (% wt)
(CH_3_)_2_	0.887	0.787
SiO	0.047	0.041
BaSO_4_	0.067	0.079
MgO	NA	0.081

**Table 3:** The mean squared error (MSE) calculated for the experimental and simulated prediction curves across the investigated energy range. The radiation shielding data are extracted from the experimental data at 12 mm.

Peak Tension (kVp)	MSE (%)		Shielding at 12 mm (%)		Pb	Pb
	ALSR-Ba	ALSR-Ba-Mg	ALSR-Ba	ALSR-Ba-Mg	0.42 mm	0.50 mm
50	2.78	1.28	1.10	0.15	0.30	0.28
60	3.58	0.18	3.21	0.30	1.17	0.38
80	2.49	0.49	4.64	3.08	4.38	2.59

## Data Availability

Data will be made available on request.
